# Metformin Decreases the Incidence of Pancreatic Ductal Adenocarcinoma Promoted by Diet-induced Obesity in the Conditional KrasG12D Mouse Model

**DOI:** 10.1038/s41598-018-24337-8

**Published:** 2018-04-12

**Authors:** Hui-Hua Chang, Aune Moro, Caroline Ei Ne Chou, David W. Dawson, Samuel French, Andrea I. Schmidt, James Sinnett-Smith, Fang Hao, O. Joe Hines, Guido Eibl, Enrique Rozengurt

**Affiliations:** 1 Department of Surgery, David Geffen School of Medicine at UCLA, Los Angeles, CA USA; 2 Department of Pathology and Laboratory Medicine, David Geffen School of Medicine at UCLA, Los Angeles, CA USA; 3 Klinik für Allgemein- und Viszeralchirurgie, Universitätsklinikum Freiburg, Freiburg, Germany; 4 Department of Medicine, David Geffen School of Medicine at UCLA, Los Angeles, CA USA

## Abstract

Pancreatic ductal adenocarcinoma (PDAC) is a particularly deadly disease. Chronic conditions, including obesity and type-2 diabetes are risk factors, thus making PDAC amenable to preventive strategies. We aimed to characterize the chemo-preventive effects of metformin, a widely used anti-diabetic drug, on PDAC development using the Kras^G12D^ mouse model subjected to a diet high in fats and calories (HFCD). *LSL-Kras*^*G12D*/+^;*p48-Cre* (KC) mice were given control diet (CD), HFCD, or HFCD with 5 mg/ml metformin in drinking water for 3 or 9 months. After 3 months, metformin prevented HFCD-induced weight gain, hepatic steatosis, depletion of intact acini, formation of advanced PanIN lesions, and stimulation of ERK and mTORC1 in pancreas. In addition to reversing hepatic and pancreatic histopathology, metformin normalized HFCD-induced hyperinsulinemia and hyperleptinemia among the 9-month cohort. Importantly, the HFCD-increased PDAC incidence was completely abrogated by metformin (*p* < 0.01). The obesogenic diet also induced a marked increase in the expression of TAZ in pancreas, an effect abrogated by metformin. In conclusion, administration of metformin improved the metabolic profile and eliminated the promoting effects of diet-induced obesity on PDAC formation in KC mice. Given the established safety profile of metformin, our findings have a strong translational potential for novel chemo-preventive strategies for PDAC.

## Introduction

Pancreatic ductal adenocarcinoma (PDAC) is a particularly aggressive and lethal cancer with an overall 5-year survival rate of approximately 8%^1^. In the United States, 53,670 new cases are expected in 2017 and PDAC presently ranks as the fourth leading cause of cancer-related mortality^1^. Due to the continuing lack of effective diagnostic and therapeutic modalities, total PDAC deaths are estimated to become the second leading cause of cancer mortality before the year 2030^2^. Research efforts have therefore increased to prevent and intercept this disease^3–5^. There is clearly an urgent need to identify novel targets and agents for prevention. Understanding the mechanisms of modifiable risk factors and repurposing currently used drugs will most likely guide the rapid implementation of new preventive strategies.

Based on epidemiological analyses, chronic conditions such as obesity and long-standing type 2 diabetes mellitus (T2DM) are linked with increased risk and worse outcomes of PDAC and other cancers^6–9^. In addition to the epidemiologic evidence, tumor-promoting effects of high-fat diets and diet-induced obesity have also been demonstrated in animal models of PDAC^10–12^. Given the increasingly high prevalence of obesity and related metabolic syndromes including T2DM, detailed mechanistic studies targeting obesity-associated PDAC are of great significance in the development of novel preventive or therapeutic strategies for this deadly disease.

Metformin (1,1-dimethylbiguanide hydrochloride) is the most widely prescribed drug for treatment of T2DM worldwide^13,14^. The primary systemic effect of metformin is the lowering of blood glucose levels through reduced hepatic gluconeogenesis and improved insulin sensitivity by increasing glucose uptake in peripheral tissues, including skeletal muscles and adipose tissue. In previous studies, we demonstrated that metformin potently stimulates AMP–activated protein kinase (AMPK) activation in PDAC cells cultured in physiological glucose^15,16^ and inhibited the mammalian target of rapamycin 1 (mTORC1) and extracellular signal-regulated kinase (ERK) via AMPK at low concentrations^15–17^. Epidemiologically, metformin administration has been linked with reduced incidence, recurrence and mortality of cancer in diabetic patients^18–25^, although a therapeutic efficacy of metformin is not universally seen in all studies^26^, especially in advanced cases of cancer. Indeed, a recent meta-analysis indicated that the effects of metformin depend on tumor stage, with marked improved survival in patients with locally advanced disease but not in patients with metastatic PDAC^23^. Although the anti-cancer activity of metformin is supported by preclinical and epidemiological studies, the mechanisms involved remain incompletely understood. Systemically, the insulin-lowering effects of metformin may contribute to its anti-cancer activity since insulin is a known mitogenic factor and hyperinsulinemia is one of the proposed mechanisms through which obesity promotes cancer. Our previous studies have demonstrated that metformin inhibits PDAC growth *in vitro* through disrupting the signaling crosstalk between G protein-coupled receptor (GPCR) and insulin/insulin-like growth factor (IGF) receptor^15,16,27^. Further, we showed that administration of metformin (given *i.p*. or via the drinking water) inhibited the growth of PDAC cells xenografted either subcutaneously or orthotopically^27,28^. However, the efficacy and mechanisms of metformin as a preventive strategy in obesity-promoted PDAC are unknown.

Previously, we have reported a highly relevant animal model, in which PDAC was promoted by diet-induced obesity (DIO). In that study, mice that express oncogenic *Kras* in the pancreas (*LSL-Kras*^*G12D*/+^*;p48-Cre:* KC) were subjected to a diet high in fats and calories (HFCD)^11,12^. When compared to lean, control diet (CD)-fed mice, HFCD-fed animals gained substantially more weight and were characterized by metabolic disturbances, including hyperglycemia, hyperinsulinemia, and hyperleptinemia. Obese KC mice developed robust pancreatic inflammation, more advanced precancer lesions, i.e. pancreatic intraepithelial neoplasia (PanIN), and higher incidence of PDAC^11,12^. In the present study, we used this animal model to characterize the chemo-preventive effects of metformin on obesity-related PDAC development. Strikingly, oral administration of metformin attenuated or even reversed the pathologic results of DIO in this model, including metabolic disturbances and increased PDAC incidence. These results suggest that metformin offers a potentially novel approach for prevention/interception of obesity-associated PDAC.

## Results

### Metformin prevents HFCD-induced weight gain, hepatic steatosis, hyperleptinemia, and hyperinsulinemia in KC mice

**Figure 1:**
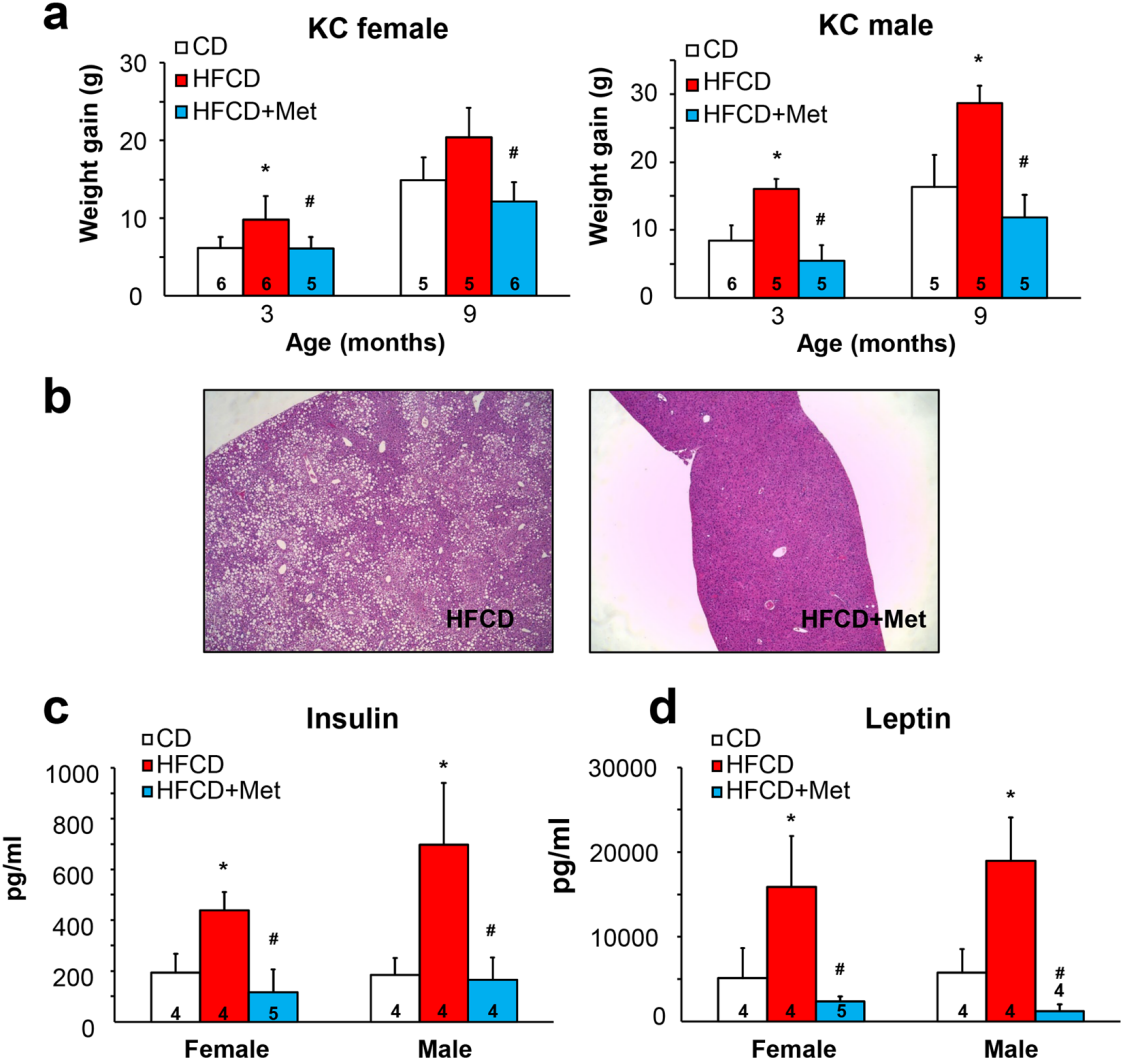
Metformin reverses weight gain, hepatic steatosis and increases in circulating insulin and leptin in KC mice subjected to a HFCD. (**a**) Weight gain (g) in female (left panel) and male (right panel) KC mice fed the CD, HFCD, or HFCD plus metformin at 3 or 9 months. Values are means ± s.d. *p < 0.05 *vs*. CD, ^#^*p* < 0.05 *vs*. HFCD. (**b**) Representative H&E staining of the liver of a KC mouse fed the HFCD (left) or HFCD plus metformin (right). (**c** and **d**) Plasma concentrations (pg/ml) of insulin (**c**) and leptin (**d**) in female and male KC mice fed the CD, HFCD, or HFCD plus metformin at 9 months. Values are means ± s.d. *p < 0.05 *vs*. CD, ^#^*p* < 0.05 *vs*. HFCD. The number of animals used in the analyses are provided in each bar.

To examine the effects of metformin on PDAC promoted by diet-induced obesity, we used a mouse model, in which an obesogenic diet markedly accelerated the development of PDAC in KC mice^11,12^. Animals were treated without or with metformin (5 mg/ml in drinking water) starting at one month until 3 or 9 months of age. This well-tolerated dose of metformin was determined based on previous reports to achieve a similar steady-state plasma concentration to that of T2DM patients^29^. The HFCD-fed KC mice gained more weight (g) than CD-fed KC mice in both females and males at 3 and 9 months. Remarkably, oral administration of metformin prevented HFCD-induced weight gain in both female and male KC mice at either 3 or 9 months (Fig. 1a).

KC mice fed the CD showed largely unremarkable liver histology with 0–5% mixed large and small droplet macro-vesicular steatosis. Obese KC mice on the HFCD showed 40–50% mixed macro- and micro-vesicular steatosis with ballooning hepatocytes. Impressively, KC mice fed the HFCD and treated with metformin displayed essentially normal liver histology with 0–5% mixed large and small droplet macro-vesicular steatosis (Fig. 1b). All mice showed minimal non-specific lymphocytic infiltrates with no significant neutrophilic inflammation in the liver. In the 3-month cohort, obese female and male KC mice displayed hyperleptinemia, which was normalized by metformin (Supplementary Fig. S1). After 3 months, insulin levels were slightly, but not significantly elevated in female and male obese KC mice (Supplementary Fig. S1). However, HFCD-fed obese 9-month-old mice developed significantly increased plasma levels of insulin and leptin, as compared with the CD-fed group (Fig. 1c and d). The increase in leptin and insulin showed a strongly positive correlation to weight gain (*p* < 0.0001). Administration of metformin prevented the increase in the levels of circulating insulin and leptin in obese female and male KC mice subjected to HFCD at 9 months (Fig. 1c and d). These results demonstrate that oral administration of metformin reversed the histologic abnormalities in the liver and prevented the hyperinsulinemia and hyperleptinemia induced by the obesogenic diet in both female and male KC mice.

### Metformin decreases the incidence of advanced PanIN-3 and the activity of proliferative signaling in HFCD-fed, obese KC mice

**Figure 2:**
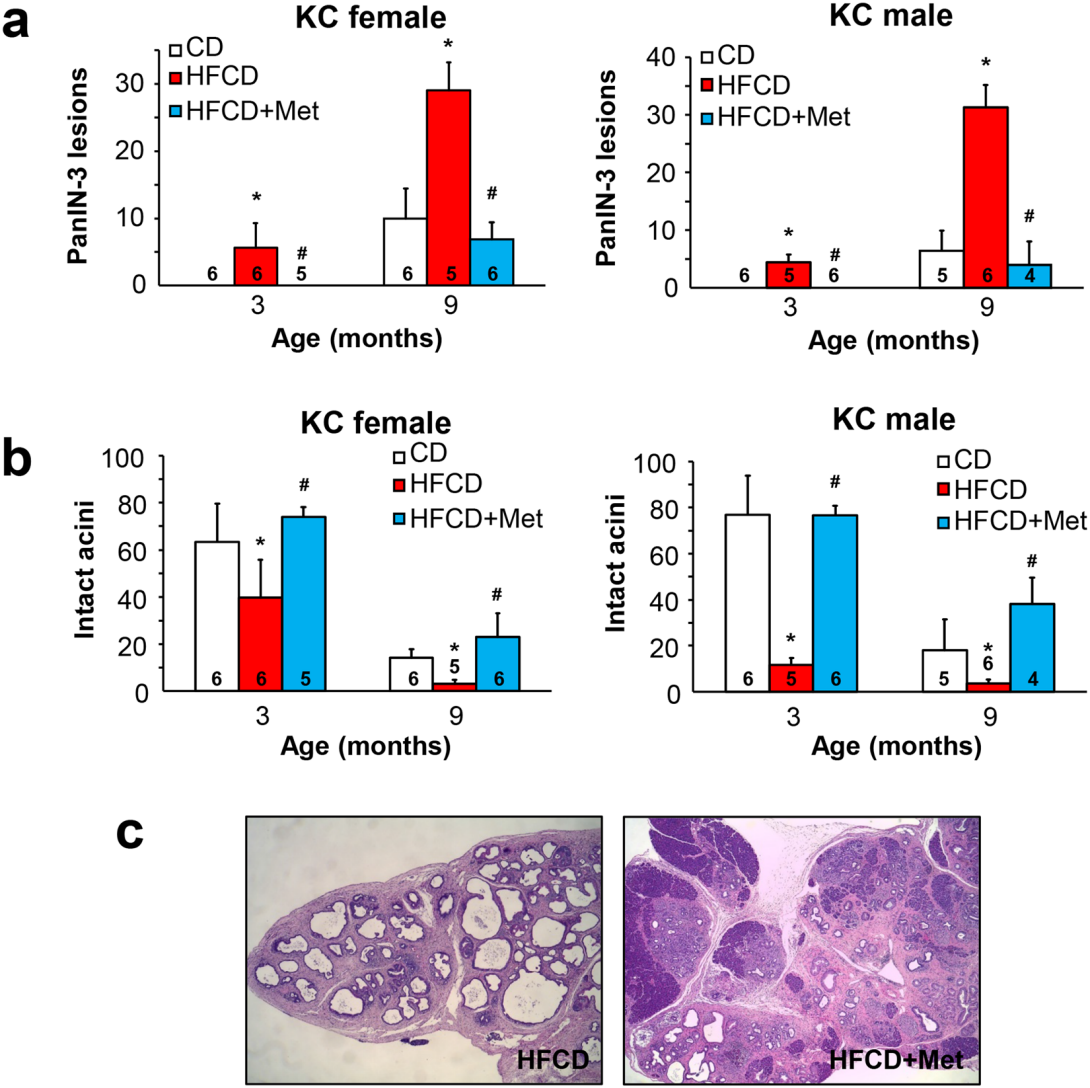
Metformin prevents the disruption in histologic pancreatic architecture induced by a HFCD in KC mice. (**a**) Percentage (%) of PanIN-3 lesions in female (left panel) and male (right panel) KC mice fed the CD, HFCD, or HFCD plus metformin at 3 or 9 months. Values are means ± s.d. *p < 0.05 *vs*. CD, ^#^*p* < 0.05 *vs*. HFCD. (**b**) Percentage (%) of intact acini in female (left panel) and male (right panel) KC mice fed the CD, HFCD, or HFCD plus metformin at 3 or 9 months. Values are means ± s.d. *p < 0.05 *vs*. CD, ^#^*p* < 0.05 *vs*. HFCD. (**c**) Representative H&E staining of the pancreas of a KC mouse fed the HFCD (left) or HFCD plus metformin (right). The number of animals used in the analyses are provided in each bar.

Next, we examined the impact of metformin on the histologic architecture of the pancreas of KC mice subjected to CD, HCFD and HFCD in conjunction with metformin. Consistent with our previous reports^11,12^, obese female or male KC mice at 3 or 9 months displayed significantly more PanIN-3, compared to age-matched lean mice. Metformin completely prevented the formation of PanIN-3 lesions in the cohort sacrificed at 3 months and drastically reduced obesity-associated PanIN-3 lesions in the 9-month cohort (Fig. 2a). Similarly, obese female or male KC mice fed the HFCD at either 3 or 9 months showed less intact acini. Administration of metformin completely prevented the depletion of intact acini at 3 months and partially reversed the striking decrease of intact acini at 9 months (Fig. 2b). Overall, the administration of metformin greatly preserved the histological architecture of the pancreas (Fig. 2c).

**Figure 3:**
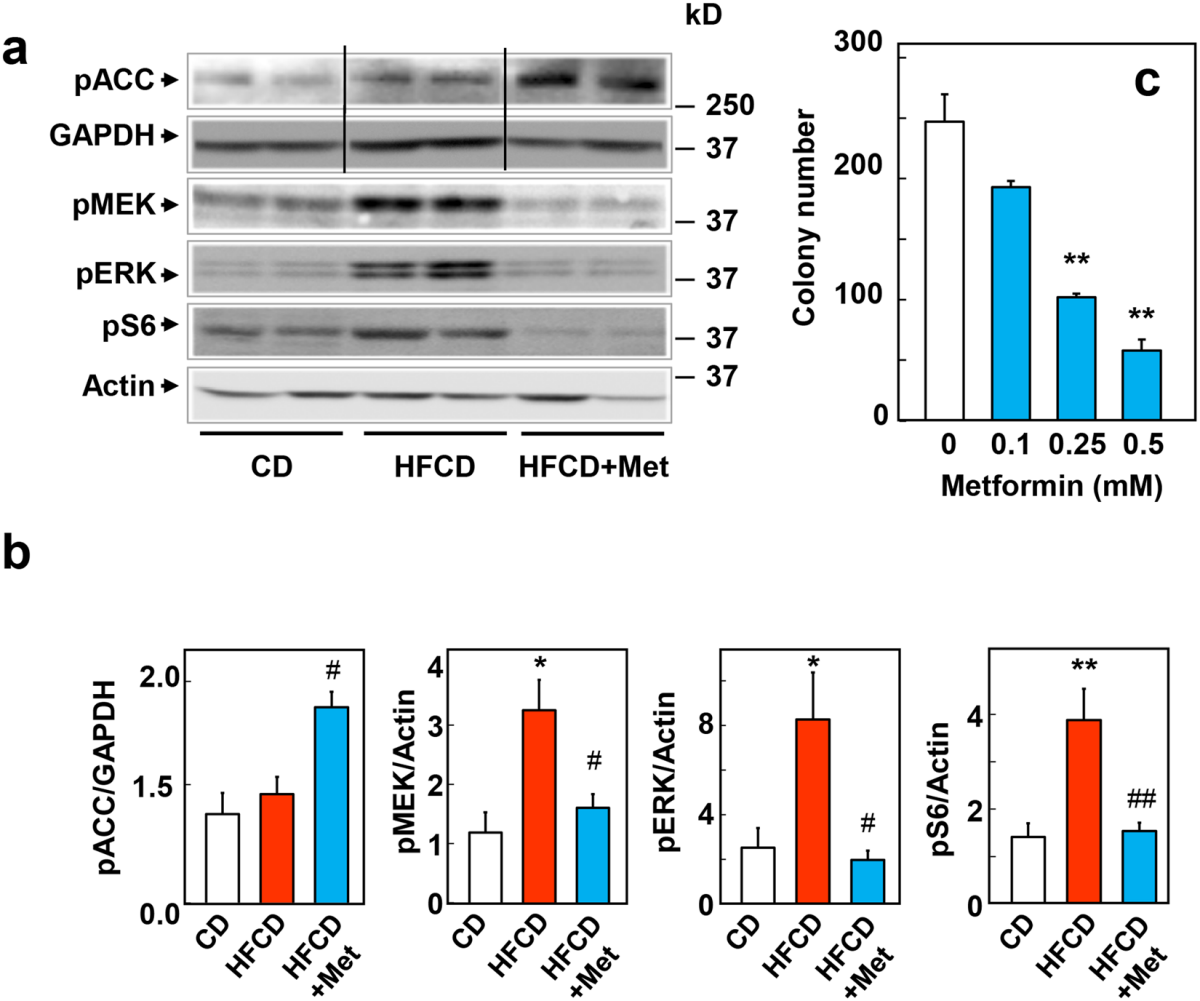
Metformin decreases the activity of proliferative signaling in HFCD-fed KC mice. (**a**) Western blotting in pancreatic lysates from KC mice fed CD, HFCD or HFCD plus metformin sacrificed at 3 months. Shown are representative immunoblots of p-ACC Ser^79^, p-MEK1/2 Ser^217/221^, p-S6 Ser^235/236^, p-ERK1/2 Thr^202^ and Tyr^204^, β-Actin and GAPDH were used as loading controls. Each lane represents an individual mouse; two mice per group are shown. For p-ACC Ser^79^ and the corresponding GAPDH loading control a cropped image is shown. Irrelevant lanes were removed (indicated by a vertical line) from the acquired digital images, and flanking lanes were juxtaposed using Adobe Photoshop. Full-length blots are presented in Supplementary Information. (**b**) Graphs show the quantification of immunoblots as ratio of phosphorylated protein to GAPDH for p-ACC Ser^79^ or β-Actin for p-MEK1/2 Ser^217/221^, p-S6 Ser^235/236^ and p-ERK1/2 Thr^202^ and Tyr^204^. Values are means ± s.e.m. n = 6. *P* values *<0.05, **<0.001 *vs*. CD; ^#^<0.05, ^##^<0.01 *vs*. HFCD. Densitometry was performed using Multi Gauge V3.0 software (Fujifilm Life Sciences) and statistical analyses conducted via SigmaPlot software. (**c**) Cell colony formation was performed as described in the Materials and Methods section. KC cells were incubated for 4 days without or with metformin at the indicated concentrations. The bars represent the number of colonies (mean ± s.e.m.; n = 3 dishes per condition). *T*-test *p* values comparing the indicated two groups to the untreated control (0) were **<0.001.

A major mechanism, by which metformin directly inhibits proliferation of PDAC cells in culture is via stimulation of AMPK^15,27,30^, which mediates inhibition of key pathways for PDAC cell proliferation, including mTORC1 and mitogen-activated protein kinase kinase (MEK)/ERK^30,31^. Consequently, we determined whether metformin increases AMPK and exerts any inhibitory effect on these pathways in the pancreas of KC mice fed the CD, HFCD, and HFCD with metformin, as scored by western blot analysis using specific antibodies to detect the phosphorylated state of these proteins in pancreatic lysates. Initially, we verified that metformin administration induced a marked increase (2.5-fold) in the phosphorylation of acetyl-CoA carboxylase (ACC) at Ser^79^ (Fig. 3a, quantification in Fig. 3b) a direct substrate of AMPK and a marker of intracellular AMPK activity^32^. These results indicate that metformin stimulates AMPK in the pancreas and are consistent with the possibility that metformin acts directly in pancreatic cells of KC mice.

Our results also show that obese KC mice displayed a marked activation of MEK (2.7-fold) and ERK (3.5-fold), as scored by phosphorylation of ERK and of MEK (Fig. 3a, quantification in Fig. 3b). Similarly, obese KC mice showed a marked increase in mTORC1 activity, as judged by the enhancement in the phosphorylation of the ribosomal protein S6 at Ser^235/236^, a direct target of p70S6 kinase (p70S6K) which in turn, is activated by mTORC1 (Fig. 3a, quantification in Fig. 3b). A salient feature of the results shown in Fig. 3a and b is that metformin administration to HFCD-fed, obese mice at 3 months profoundly inhibited the increase in the phosphorylation of MEK, ERK, and S6 Ser^235/236^.

In order to explore further whether metformin can act directly on pancreatic cells of mice harboring *Kras*^G12D^, we determined the ability of metformin to inhibit colony formation of cells isolated from KC mice and cultured in medium containing a physiological concentration of glucose^15,16^. As shown in Fig. 3c, metformin significantly inhibited colony formation at low concentrations in KC cells, with 50% inhibition of colony formation produced at ~0.2 mM.

### Metformin decreases the incidence of PDAC and prevents the increase in TAZ expression in KC mice subjected to HFCD

**Figure 4:**
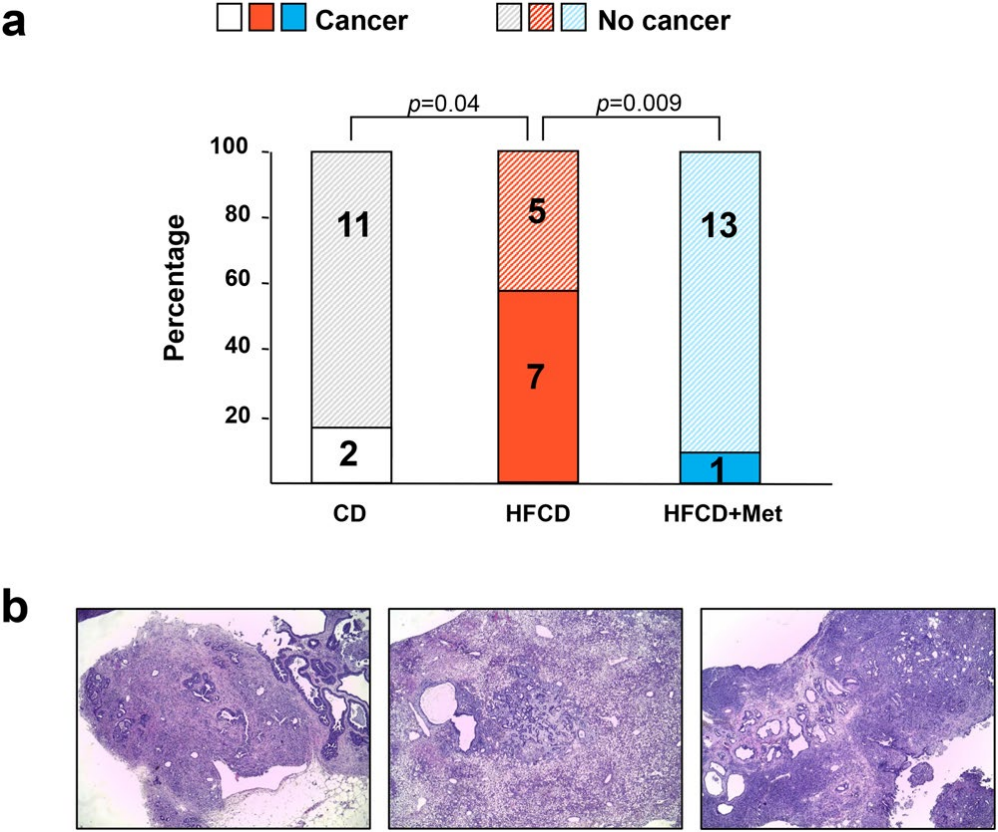
Metformin reverses the increase in PDAC incidence in HFCD-fed KC mice. **(a)** PDAC incidence (%) in KC mice fed the CD, HFCD, or HFCD with metformin and sacrificed at 9 months of age. Values in bars denote numbers of mice without or with PDAC. **(b)** Representative H&E images of PDAC (left and right panel) in KC mice fed the HFCD and a liver metastasis (center) in a KC mouse fed the HFCD.

Subsequently, we determined the cancer incidence in lean and obese KC mice subjected to CD, HFCD or HFCD with metfomin (Fig. 4). PDAC was identified in 2 of 13 mice (15%) fed the CD but in 7 of 12 mice (58%; 5 of 7 male and 2 of 5 female mice) subjected to HFCD (CD vs. HFCD, *p* = 0.04), in line with recent results showing that an obesogenic diet markedly increases the incidence of PDAC in KC mice^11^. A striking feature of the results presented in Fig. 4 is that only 1 out of 14 mice (7%; 1 of 8 females) treated with HFCD and metformin at 9 months developed PDAC (HFCD vs. HFCD + metformin, *p* < 0.01). The data clearly indicate that metformin administration dramatically reduced the incidence of advanced PanIN-3 (Fig. 2) and PDAC (Fig. 4) in obese, HFCD-fed KC mice.

**Figure 5:**
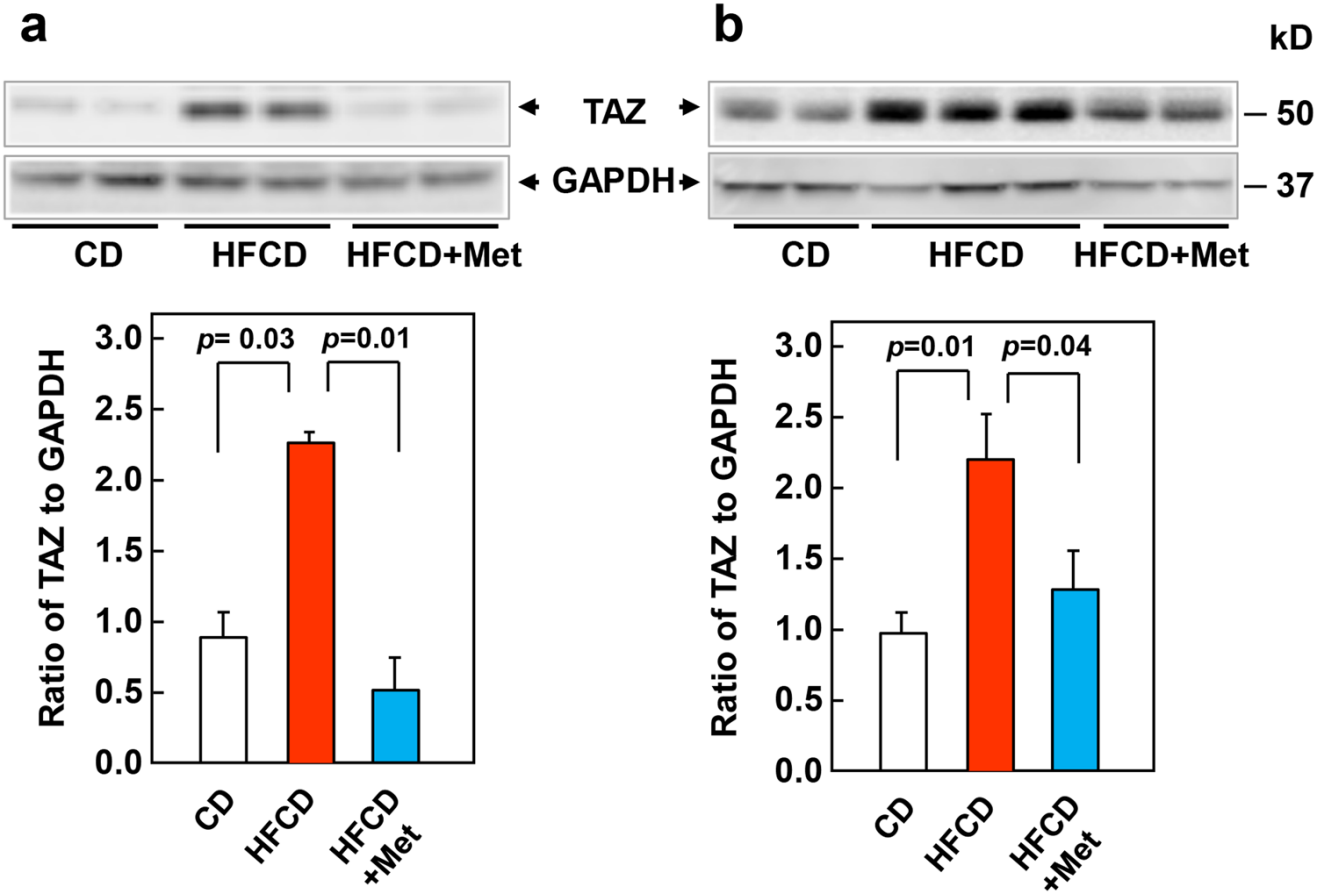
Metformin prevents the increase in the expression of TAZ protein in pancreatic lysates of KC mice subjected to a HFCD. Western blotting in pancreatic lysates from KC mice fed the CD or HFCD or HFCD plus metformin (HFCD + Met) at 3 months (**a**) and 9 months (**b**). Shown are representative immunoblots of TAZ and GAPDH, used as loading control. Each lane represents an individual mouse. Bar Graphs show the ratio of TAZ protein to GAPDH, mean ± s.e.m., n = 6. Densitometry was performed using Multi Gauge V3.0 software (Fujifilm Life Sciences) and statistical analyses conducted via SigmaPlot software. Uncropped full-length blots are presented in Supplementary Information.

The transcriptional co-activator WW-domain-containing Transcriptional co-Activator with PDZ-binding motif (TAZ), a major downstream effector of the Hippo pathway, has emerged as key oncogenic protein in PDAC^33–35^ and other cancer cells^36^. TAZ and its paralog Yes-associated protein (YAP), act downstream of KRAS signaling in PDAC^33,34^. TAZ differs from YAP, the other major effector of the Hippo pathway, in its protein stability. TAZ is an unstable protein that contains N-terminal and C-terminal phosphodegrons^37,38^ leading to proteolytic degradation and thus, the level of TAZ protein expression is a marker of its activity in the cell^39^. Consequently, we determined whether the expression of TAZ increases in the pancreas of KC mice subjected to HFCD and whether metformin administration has any effect on the levels of this oncogenic protein. Western blot analysis demonstrated a marked increase in the levels of TAZ protein in pancreatic extracts of KC mice subjected to HFCD at either 3 or 9 months, as compared with lean KC mice on CD (Fig. 5). A noticeable aspect of the data is that the administration of metformin, under conditions that prevented the formation of PanIN-3 lesions and depletion of intact acini (Fig. 2) and PDAC development (Fig. 4), virtually abrogated the increase in the expression of TAZ protein.

## Discussion

It is of great interest to explore novel interventional strategies for PDAC, an almost universally lethal disease with few efficacious treatment modalities. Given the high prevalence of obesity and its role in promoting cancer development, targeting the interplay of obesity, diabetes, and PDAC will shed new light on treatment options. In particular, repurposing metformin for the prevention of obesity-associated PDAC has great clinical relevance. In the present study, we demonstrate that orally administered metformin significantly prevents the development of PDAC in a relevant mouse model characterized by obesity and insulin resistance.

In our animal model, the HFCD led to a marked increase of weight gain and elevated plasma leptin levels evident by 3 months of age, and significantly higher plasma insulin levels at a later time point (9 months of age). The observed hyperinsulinemia and hyperleptinemia were highly correlated with HFCD-induced weight gain, recapitulating conditions in most overweight or obese individuals who have at least some degree of insulin and leptin resistance. Importantly, the HFCD-induced weight gain and metabolic disturbance were completely prevented by the intake of metformin, which is consistent with its documented effects^40^. These results also indicate that biologically effective concentrations of metformin were achieved in those mice receiving the drug.

Another prominent feature of the HFCD-fed mice is hepatic steatosis. This condition is within the spectrum of non-alcoholic fatty liver disease (NAFLD) that affects 60–95% of obese population, and is strongly associated with insulin resistance^41^. Insulin resistance has been proposed as a central mechanism leading to hepatic steatosis^42^, thereby making insulin-sensitizing drugs such as metformin a potential therapeutic option for this liver disease. Our study shows that metformin ameliorated hepatic steatosis associated with HFCD-induced obesity, which is consistent with other reports using high-fat diet-fed experimental animals^43–45^. However, a meta-analysis, which included only a few randomized trials with small patient numbers, concluded that 4–12 months of metformin use has no significant beneficial effect on improving liver histology in NAFLD patients^46^. Our results may support future larger randomized, placebo-controlled trials (RCTs) evaluating higher dose, long-term efficacy, and early intervention in the disease course, as well as identifying the most susceptible population, to achieve the maximal beneficial impact of metformin.

Despite accumulating epidemiological evidence indicating the anti-cancer effects of metformin, it remains controversial whether metformin use is associated with improved PDAC outcome^47^. In particular, metformin seems to offer no significant survival benefits in the late stage disease, based on two recently published RCTs examining the effect of metformin on overall survival in patients with advanced PDAC^23,48,49^. However, a recent multicenter, double-blind RCT investigated the chemo-preventive efficacy of metformin in colorectal cancer and found that low-dose metformin for one year significantly reduced the incidence of recurrent polyps and adenomas^50^, implicating that the use of metformin in cancers is likely more applicable in preventive rather than therapeutic settings. Unlike colorectal cancer, evidence for the chemo-preventive property of metformin in PDAC has not been demonstrated by randomized trials and is largely derived from normal weight/non-diabetic animal models^51–53^, where metformin might be less effective^54,55^.

To the best of our knowledge, the anti-neoplastic effects of metformin on PDAC in the context of diet-induced obesity with insulin resistance has not yet been reported in genetically engineered animal models of PDAC. In line with previous studies using high-fat diet-fed, carcinogen-induced^56^ and engrafted^55,57^ PDAC models, our results show that metformin effectively prevents the progression of advanced PanINs and the development of KRAS-driven PDAC promoted by diet-induced obesity.

The anti-cancer property of metformin can be mediated by direct, indirect (systemic) or combined effects of the drug, and it has been a major challenge to differentiate these mechanisms *in vivo*. Interestingly, HFCD-induced histological changes, including PanIN-3 formation and acini depletion, were completely restored by metformin at 3 months, when its insulin-lowering effect was not significant. This observation suggests that, at least in the model used in this study, the mechanism through which metformin exerts its anti-neoplastic activity involves more direct effects on transformed cells. In line with this possibility, we found that metformin produced a marked increase in AMPK activity within the pancreas, as scored by phosphorylation of ACC at Ser^79^, a site directly phosphorylated by AMPK and consequently a marker of the activity of the enzyme in the cell^32^. Furthermore, metformin also inhibited the activation of other pathways targeted by AMPK, including mTORC1 and MEK/ERK, in pancreatic lysates of KC mice receiving an obesogenic diet. Collectively, these results are consistent with the notion that the cancer preventive effects of metformin are mediated, at least in part, through direct effects on pancreatic epithelial cells harboring *Kras*^G12D^.

The transcriptional co-activators YAP and TAZ, two major downstream effectors of the Hippo pathway implicated in driving multiple cancers^36^, have emerged as key oncogenic proteins in PDAC that act downstream of KRAS signaling^33,34^. There are several possible pathways, by which KRAS regulates YAP/TAZ localization and activity, including Rho, PI3K, PKD and GPCRs^58^. TAZ differs from YAP, the other major effector of the Hippo pathway, in its protein stability. Accordingly, the level of these oncogenic proteins often are differentially expressed in different cancer cell types^59^. TAZ is an unstable protein that contains N-terminal and C-terminal phosphodegrons. The phosphorylation of the two phosphodegrons mediated by large tumor suppressor kinase 1/2 (LATS1/2) and nutrient-sensitive kinases, including phosphoinositide 3-kinase (PI3K)/ protein kinase B (AKT)/ glycogen synthase kinase 3 (GSK3)^37,38^ promotes proteasome-mediated degradation and thereby regulates TAZ protein expression and activity in the cell^39^. However, it was not known whether an obesogenic diet that promotes PDAC development in KC mice has any effect on the level of pancreatic TAZ. In this study, we found a marked increase in the level of TAZ protein in pancreatic extracts of KC mice subjected to HFCD. Shown for the first time, the administration of metformin, under conditions that prevented the formation of PanIN-3 lesions, depletion of intact acini and PDAC development, virtually abrogated the increase in the expression of TAZ protein. It is plausible that the suppressing effects of metformin on TAZ expression are mediated by the inhibitory effects of metformin on obesogenic signaling or by direct inhibitory actions of AMPK, which is activated by metformin, on YAP/TAZ^58^. Given recent reports implicating TAZ in PDAC development^33–35^, it is possible that HFCD-induced TAZ protein accumulation is one of the mechanisms by which the obesogenic diet promotes and metformin opposes PDAC development in KC mice.

In conclusion, our results support the notion that metformin, the most widely prescribed drug for treatment of T2DM worldwide, could be chemo-preventive for PDAC particularly in the obese population with new-onset diabetes mellitus that is at higher risk of developing PDAC.

## Methods

### Experimental animals

Offspring of *LSL-Kras*^*G12D*/+^ and *p48-Cre*^+/−^ mice^60^ were randomly allocated to a control diet (CD), a high fat, high calorie diet (HFCD), or HFCD with metformin in the reverse osmosis drinking water (5 mg/ml) at one month of age. All mice had free access to the diet. Mice fed the HFCD has free access to either regular drinking water or drinking water (reverse osmosis) supplemented with metformin (5 mg/ml). Water supplemented with metformin was made fresh and replenished twice weekly. Body weights were measured weekly and the general health and behavior of animals were assessed daily. At 3 and 9 months of age cohorts of mice (male and female) were sacrificed and tissues harvested. All studies involving animals were reviewed and approved by the Chancellor’s Animal Research Committee of the University of California, Los Angeles in accordance with the NIH Guide for the Care and Use of Laboratory Animals (protocol number: 2011-118).

### Genotyping analysis

*LSL-KRAS*^*G12D*^ and *Cre* alleles were detected prior to randomizing to the experimental diets by PCR analysis of genomic DNA, as described^61^. Mutant (KC) mice expressed both *LSL-KRAS*^*G12D*^ and *Cre* alleles, and animals carrying neither allele were labeled wildtype (WT).

### Experimental diets

Diets were prepared by Dyets, Inc. (Bethlehem, PA). At one month of age mice were randomized to receive either the CD, HFCD, or HFCD with 5 mg/ml metformin (Sigma-Aldrich, St. Louis, MO) in the drinking water. A detailed composition of the diets was described previously^12^. Briefly, while the CD contained 12% calories from fat, 40% of calories in the HFCD stem from fat (corn oil-based). All diets were stored at −20 °C (long-term) or 4 °C (short-term) in sealed containers and prepared under low light conditions. The diets were replaced on a weekly basis and the metformin containing water was refreshed twice a week.

### Metabolic panel

At the end of the study, blood was collected by cardiac puncture. Plasma was obtained by centrifugation (5,000 rpm for 10 minutes at room temperature) and stored at −80 °C. Levels of insulin and leptin were quantified using the MILLIPLEX MAP Mouse Adipokine Magnetic Bead Panel - Endocrine Multiplex Assay (EMD Millipore, Billerica, MA) according to the manufacturer’s guidelines. Cholesterol, glucose, and triglycerides were measured by the UCLA Division of Laboratory Animal Medicine.

### Pancreas and liver histology

Hematoxylin and eosin (H&E) stained tissue sections of the pancreas and liver, fixed in formalin and embedded in paraffin, were assessed by gastrointestinal pathologists blinded to the experimental groups. The presence and stage of murine PanINs and PDACs were analyzed according to previously published criteria^62,63^. For each animal approximately 100 pancreatic ducts (tail of the pancreas) were quantified and the proportion of murine PanIN-3 to the overall number of pancreatic ducts was recorded.

### Colony Formation

KC cells were grown in Dulbecco Modified Eagle Medium (DMEM) with 2 mmol/L glutamine, 1 mmol/L Na-pyruvate, 100 U/mL penicillin, and 100 μg/mL streptomycin and 5% fetal bovine serum (FBS) at 37 °C in a humidified atmosphere containing 10% CO_2_. For cell colony formation, 500 KC cells were plated into 35-mm tissue culture dishes in DMEM containing 5% FBS. After 24 hours of incubation at 37 °C, cultures were switched to DMEM containing 1% FBS either in the absence or presence of metformin. A colony consisted of at least 50 cells. Cell colony numbers from three dishes per condition were determined after 4 days of incubation.

### Western blotting

Tissue samples were homogenized in G-Lysis (Cytoskeleton, Inc., Denver, CO) buffer and the resulting homogenates were centrifuged at 18,000 × g. The protein concentration of the supernatants was determined with the bicinchoninic acid (BCA) Protein Assay kit (Thermo Fisher Scientific, Waltham, MA). An equal volume of 4× sodium dodecyl sulfate (SDS)-polyacrylamide gel electrophoresis (PAGE) sample buffer (40 mM Tris/HCl, pH 6.8, 6% SDS, 4 mM ethylenediaminetetraacetic acid (EDTA), 8% 2-mercaptoethanol, 20% glycerol) was added to each supernatant and boiled for 10 min, followed by SDS-PAGE on 4–15% gradient gels and transfer to Immobilon-P membranes (EMD Millipore). Western blots were performed on membranes incubated overnight with the specified antibodies in PBS containing 0.1% Tween-20. The immunoreactive bands were detected with enhanced chemiluminescence (ECL) reagents (GE Healthcare Bio-Sciences Corp, Piscataway, NJ). The membranes were imaged using a chemiluminescence image analyzer LAS-4000 mini (Fujifilm Life Sciences, Tokyo, Japan), and the intensity of signals normalized to the loading controls was quantified with Multi Gauge V3.0 software (Fujifilm Life Sciences). The antibodies used were: polyclonal antibodies to phospho acetyl-CoA carboxylase Ser^79^, phospho MEK1/2 Ser^217/221^, phospho S6 Ser^235/236^, and a monoclonal antibody to phospho ERK1/2 Thr^202^ and Tyr^204^, and YAP/TAZ, all obtained from Cell Signaling Technology (Danvers, MA). Equal loading of the gels was verified using monoclonal antibodies to β-Actin and or glyceraldehyde 3-phosphate dehydrogenase (GAPDH) (Santa Cruz Biotechnology, Santa Cruz, CA).

### Statistical analysis

Values are presented as mean ± standard deviation (s.d.) or standard error of mean (s.e.m.). In general, one-way (or two-way) ANOVA and two-tailed Student’s *t*-tests assuming unequal variances were performed to assess statistical significance. Significance was considered at a *p-*value less than 0.05 and indicated by an asterisk (*). For the comparison of cancer incidences, Fisher’s exact test was performed.

### Data availability

All data generated or analyzed during this study are included in this published article (and its Supplementary Information file).

## Electronic supplementary material

Supplementary information
