# Supplementary material for: Metformin Decreases the Incidence of Pancreatic Ductal Adenocarcinoma Promoted by Diet-induced Obesity in the Conditional KrasG12D Mouse Model
Source: Sci Rep. 2018 Apr 12;8:5899. doi: 10.1038/s41598-018-24337-8 (PMC5897574; doi:10.1038/s41598-018-24337-8)
Supplement: Supplementary file 1 — Supplementary information [file 41598_2018_24337_MOESM1_ESM.pdf]

Metformin Decreases the Incidence of Pancreatic Ductal Adenocarcinoma Promoted by Diet-induced Obesity in the Conditional KrasG12D Mouse Model

Hui-Hua Chang, Aune Moro, Caroline Ei Ne Chou, David W. Dawson, Samuel French, Andrea I. Schmidt, James Sinnett-Smith, Fang Hao, O. Joe Hines, Guido Eibl*, and Enrique Rozengurt* (* Dual senior authorship).

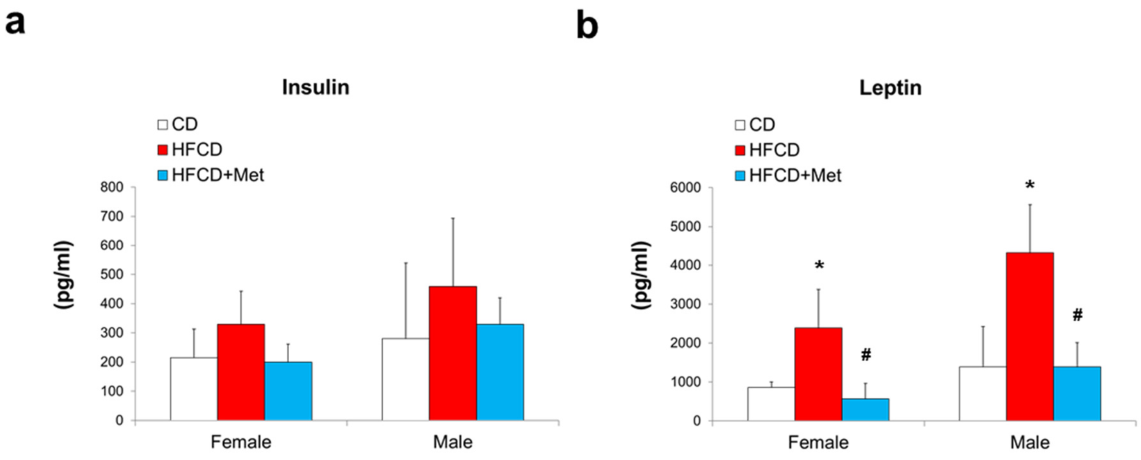

Supplementary Figure S1. Plasma concentrations (pg/ml) of insulin **(a)** and leptin **(b)** in female and male KC mice fed the CD, HFCD, or HFCD plus metformin (5 mg/ml in drinking water) at 3 months. Values are mean ± s.d. *;*p*<0.05 *vs.* CD, #;*p*<0.05 *vs.* HFCD.

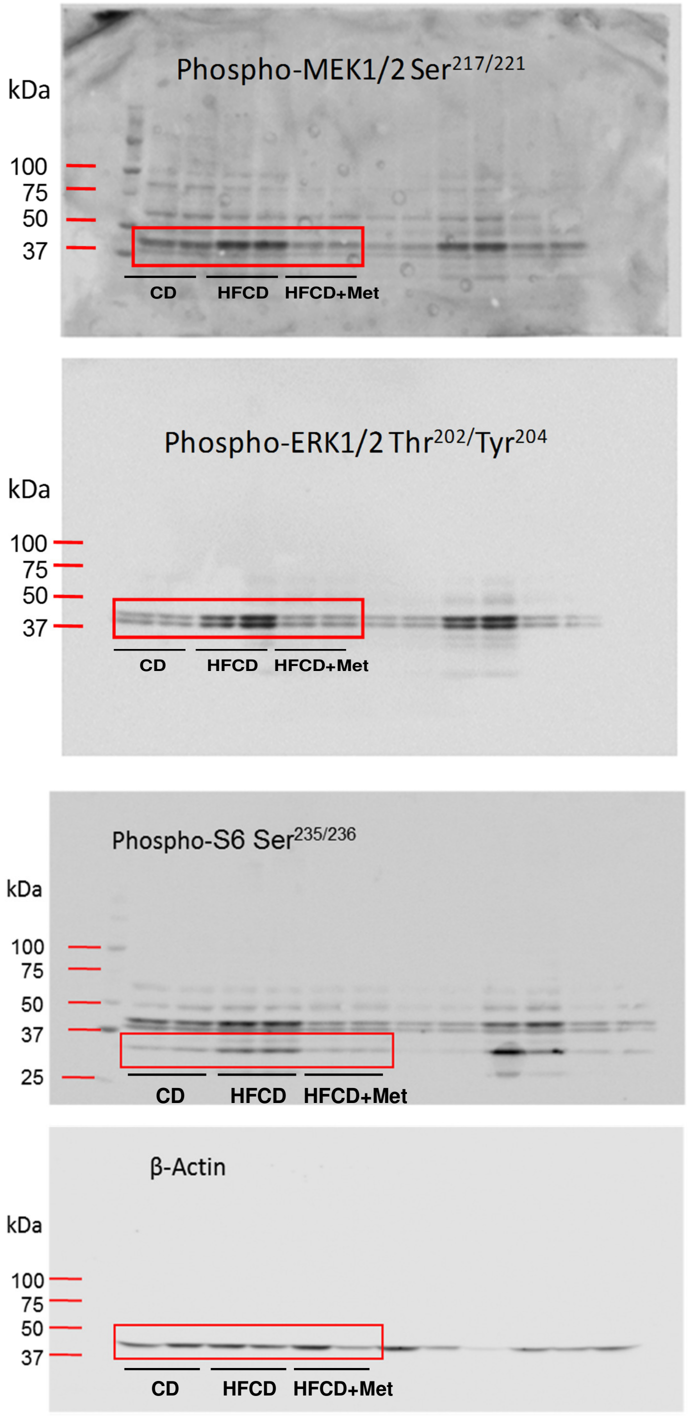

Supplementary Figure S2. Uncropped images for Figure 3a (Part A). Red outlines represent lanes shown in **Figure 3a**. All immunoblots were visualized via an LAS-4000 mini chemiluminescence image analyzer (Fujifilm Life Sciences, Tokyo, Japan).

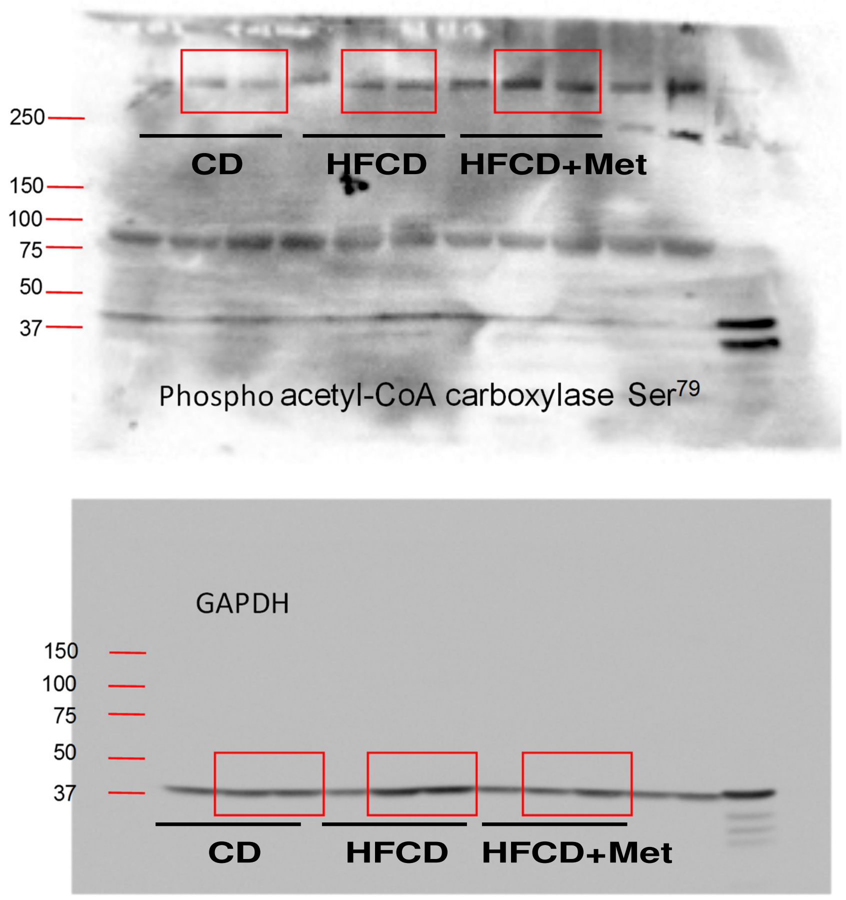

Supplementary Figure S2. Uncropped images for Figure 3a (Part B). Red outlines represent lanes shown in **Figure 3a**. All immunoblots were visualized via an LAS-4000 mini chemiluminescence image analyzer (Fujifilm Life Sciences, Tokyo, Japan).

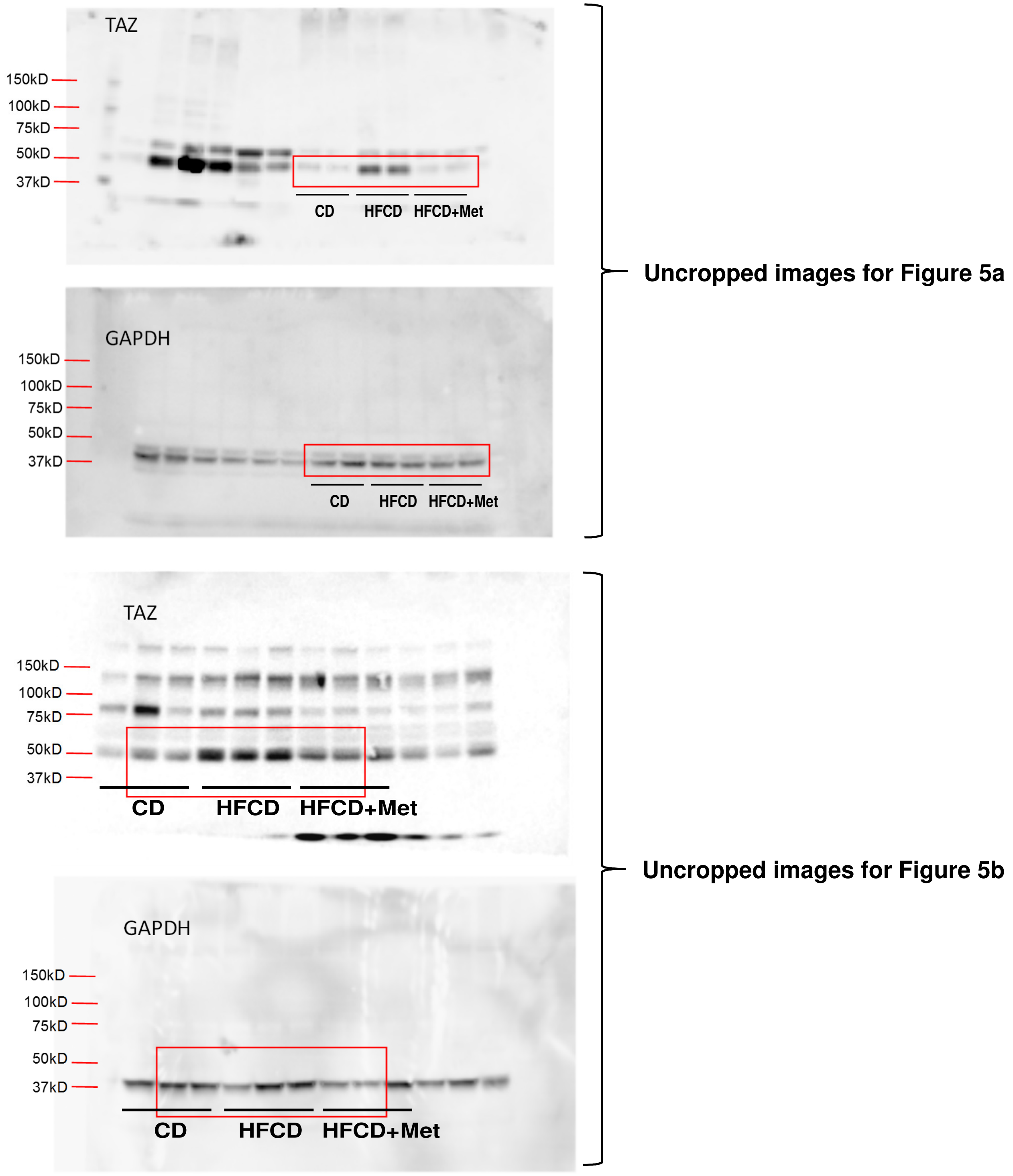

Supplementary Figure S3. Uncropped images for Figure 5a and 5b. Red outlines indicate lanes shown in **Figure 5a** and **5b**. All immunoblots were visualized via an LAS-4000 mini chemiluminescence image analyzer (Fujifilm Life Sciences, Tokyo, Japan).
